# Crystal structure and Hirshfeld analysis of *trans*-bis­(5-fluoro­indoline-2,3-dione 3-oximato-κ^2^ *O* ^2^,*N* ^3^)-*trans*-bis­(pyridine-κ*N*)copper(II)

**DOI:** 10.1107/S2056989018003365

**Published:** 2018-03-02

**Authors:** Ana Paula Lopes de Melo, Leandro Bresolin, Bianca Barreto Martins, Vanessa Carratu Gervini, Adriano Bof de Oliveira

**Affiliations:** a Escola de Química e Alimentos, Universidade Federal do Rio Grande, Av. Itália km 08, Campus Carreiros, 96203-900 Rio Grande-RS, Brazil; b Departamento de Química, Universidade Federal de Sergipe, Av. Marechal Rondon s/n, 49100-000 São Cristóvão-SE, Brazil

**Keywords:** crystal structure, 5-fluoro­isatin 3-oxime copper complex, Hirshfeld surface analysis

## Abstract

The reaction in methanol of Cu^II^ acetate monohydrate with 5-fluoro­isatin 3-oxime deprotonated with KOH in a 1:2 molar ratio and recrystallization from pyridine yielded the title compound, [Cu(C_8_H_4_FN_2_O_2_)_2_(C_5_H_5_N)_2_]. In the centrosymmetric complex, the anionic form of the isatin oxime acts as a κ^2^ *N*,*O* donor, building five-membered metallarings. The Cu^II^ cation is sixfold coordinated in a slightly distorted octa­hedral environment by two *trans*, equatorial, anionic isatin derivatives and two *trans* pyridine ligands in axial positions. The complexes are linked by hydrogen bonding into a three-dimensional network, which is also stabilized by π–π stacking inter­actions [centroid-to-centroid distance = 3.7352 (9) Å] and C—H⋯π contacts. The Hirshfeld surface analysis indicates that the major contributions for the crystal packing are H⋯H (31.80%), H⋯C (24.30%), H⋯O (15.20%) and H⋯F (10.80%). This work is the second report in the literature of a crystal structure of a coordination compound with isatin 3-oxime ligands (coordination chemistry).

## Abstract 2

The crystal structure and the Hirshfeld surface analysis of a 5-fluoro­isatin 3-oxime and copper(II) complex are reported. In the crystal, the centrosymmetric complexes are linked by hydrogen bonding into a three-dimensional network. This work is the second report in the literature of a crystal structure with isatin 3-oxime derivatives acting as ligands (for metal complexes).

## Chemical context   

By the first half of the 19th century, the first reports on the chemistry of the isatin fragment were published independently in Germany and France (Erdmann, 1841*a* ▸,*b* ▸; Laurent, 1841 ▸). One very nice review concerning the organic synthesis of the isatin derivatives was published 74 years ago (Sumpter, 1944 ▸) and the topic remains up-to-date. From the early years, the chemistry of isatin-based mol­ecules emerged from the synthetic approach to a large class of organic compounds with applications in biochemistry and pharmacology. For two recent examples, see: 1-[(2-methyl­benzimidazol-1-yl) meth­yl]-2-oxo-indolin-3-yl­idene]amino]­thio­urea, a derivative with *in silico* and *in vitro* inhibition of Chikungunya virus replication (Mishra *et al.*, 2016 ▸) and 5-chloro­isatin-4-methyl­thio­semi­carbazone, another derivative which appears as an inter­mediate in the synthesis of an HIV-1 RT inhibitor (Meleddu *et al.*, 2017 ▸). The abbreviation HIV-1 RT stands for human immunodeficiency virus type 1 reverse transcriptase. Along the same line of research of the present work, the crystal structure, the Hirshfeld surface analysis and the lock-and-key supra­molecular analysis through *in silico* evaluation with the vascular endothelial growth factor receptor-2 (VEGFR-2) of the isatin derivative ligand of the title complex were recently carried out. The (3*Z*)-5-fluoro-3-(hy­droxy­imino)-indolin-2-one mol­ecule showed a structure–activity relationship with the selected biological target through hydrogen bonding (Martins *et al.*, 2017 ▸). Although the chemistry of isatins is already well reported in several scientific disciplines, crystal structures of complexes with isatin 3-oxime derivatives are surprisingly few in number. Thus, the crystal structure determination of isatin-based mol­ecules has become our major research inter­est and herein, the synthesis, crystal structure and Hirshfeld surface analysis of a 5-fluoro­isatin 3-oxime complex with copper(II) is reported.
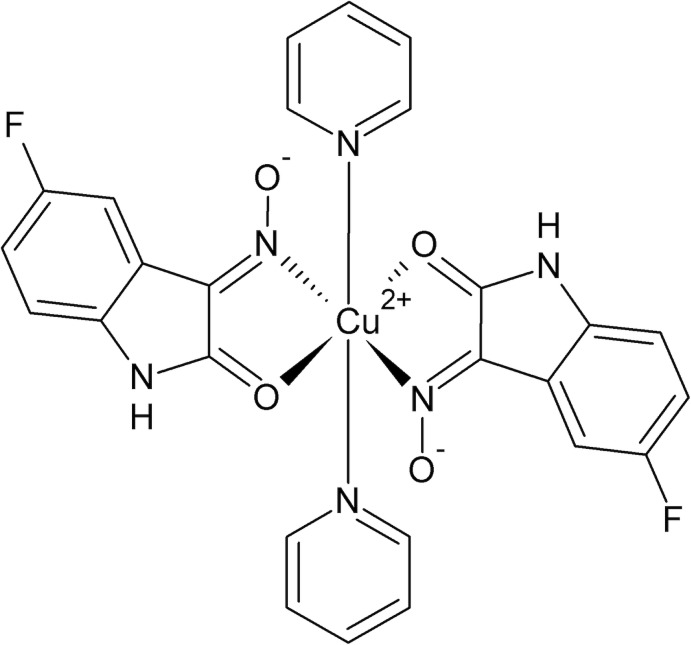


## Structural commentary   

**Figure 1:**
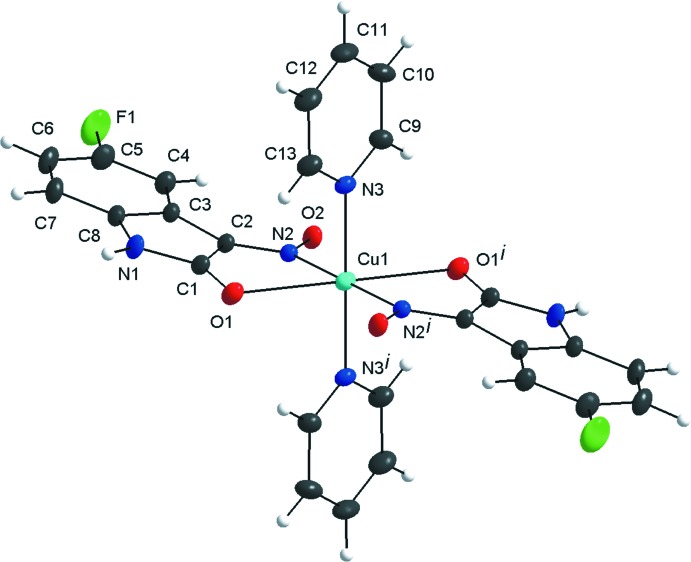
The mol­ecular structure of the title compound, showing the atom labelling and displacement ellipsoids drawn at the 40% probability level. [Symmetry code: (i) −*x* + 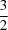, −*y* + 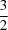, −*z* + 1.]

**Figure 2:**
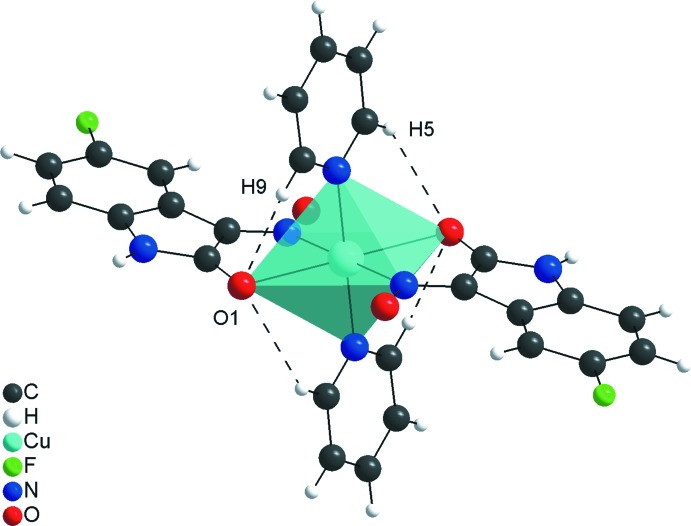
The intra­molecular C—H⋯O hydrogen inter­actions of the title compound (dashed lines) forming a ring of *S*(5) graph-set motif.

The asymmetric unit of the title coordination compound consists of one Cu^II^ cation, which lies on an inversion center, and two ligands in general positions, the anionic form of 5-fluoro­isatin 3-oxime and one pyridine mol­ecule. The Cu^II^ atoms are sixfold coordinated in a slightly distorted octa­hedral environment by two five-membered chelate 5-fluoro­isatin-3-oximate ligands, acting as *κ* ^2^ *N*,*O*-donors in equatorial positions, and by two pyridine ligands in axial positions (Fig. 1 ▸). The isatin 3-oxime derivative is nearly planar with an r.m.s. deviation from the mean plane of the non–H atoms of 0.0145 Å and a maximum deviation of 0.0344 (9) Å for the N2 atom. The dihedral angle between the pyridine ring and the mean plane through the indoline ring system is 73.82 (3)°. For the five-membered ring, the r.m.s. from the mean plane through the Cu1/C1/C2/N2/O1 fragment is 0.074 Å and the maximum deviation from that plane is 0.0945 (7) Å for the N2 atom. The N2—Cu1—N3 and O1—Cu1—N3 angles are 88.75 (4) and 89.01 (4)°, respectively. Four intra­molecular C—H⋯O hydrogen bonds are observed for the title compound, forming rings with *S*(5) graph-set motif. As an inter­esting feature of the structure, a hydrogen-bonded macrocyclic coordination environment can be assumed based on the *S*(5) rings (Fig. 2 ▸, Table 1 ▸).

## Supra­molecular features and Hirshfeld analysis   

**Figure 3:**
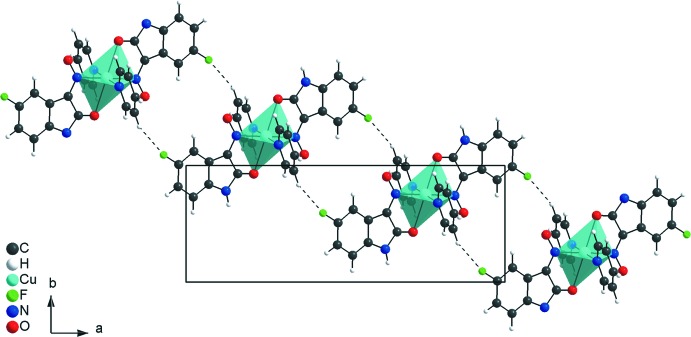
Partial crystal packing of the title compound, viewed down the *c* axis, showing the C—H⋯F inter­actions (dashed lines) forming rings of 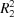
(22) graph-set motif connecting the mol­ecules into a chain along the [110] direction.

**Figure 4:**
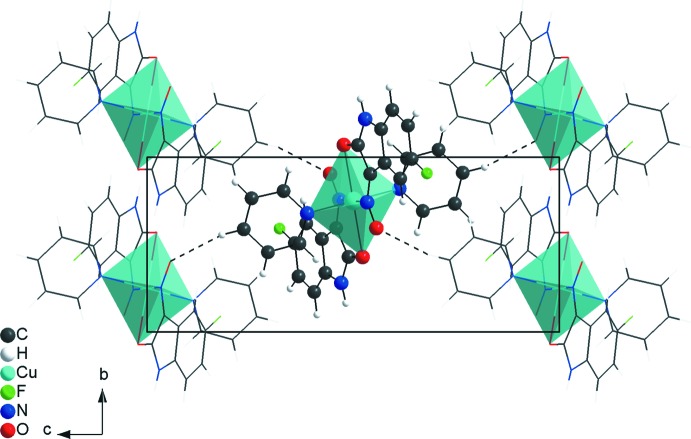
Partial crystal packing of the title compound, viewed down the *a* axis, showing the C—H⋯O inter­actions (dashed lines) organized in a *C*(8) graph-set motif along the [001] direction.

**Figure 5:**
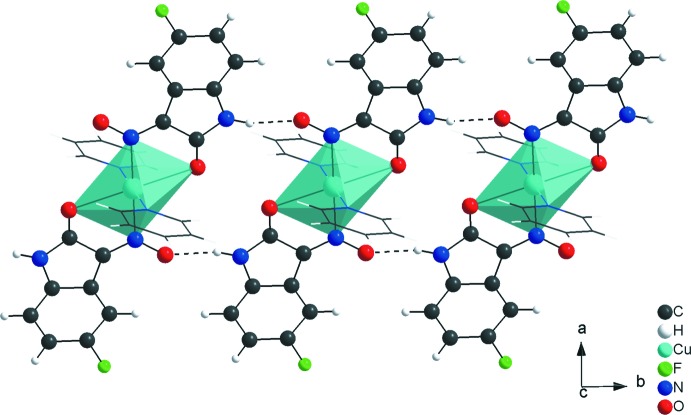
Partial crystal packing of the title compound, viewed along the *c* axis, showing the C—H⋯O inter­actions (dashed lines) forming rings of 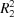
(14) graph-set motif connecting the mol­ecules into a chain along the [010] direction.

In the crystal, the mol­ecules of the centrosymmetric title compound are connected into a three-dimensional hydrogen-bonded network (Table 1 ▸). The complexes are linked by centrosymmetric pairs of C—H⋯F inter­actions into dimers with graph-set motif *R_2_^2^*(22). The dimers are the subunits of the periodic arrangement along the [110] direction (Fig. 3 ▸). The mol­ecular units are also connected by C—H⋯O inter­actions into a one-dimensional hydrogen-bonded polymer along the [001] direction (Fig. 4 ▸) and finally, the complexes are linked by N—H⋯O inter­actions into centrosymmetric dimers with graph-set motif *R_2_^2^*(14). Like the dimers of the first structural element, with C—H⋯F inter­actions connecting the mol­ecules, the latter element is also based on dimers as subunits of the polymeric motif, connected through N—H⋯O inter­actions but in this case along the [010] direction (Fig. 5 ▸). In addition, π–π stacking ­inter­actions [centroid-to-centroid distance: 3.7352 (9) Å] and C—H⋯π contacts (Table 1 ▸) stabilize the crystal structure.

**Figure 6:**
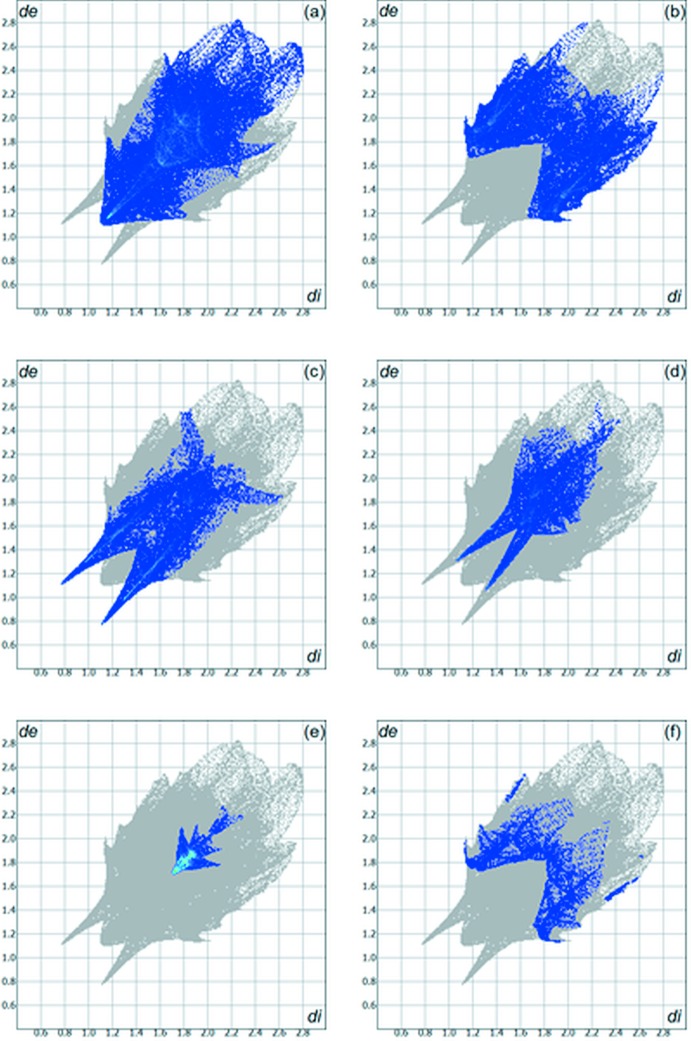
Hirshfeld surface two-dimensional fingerprint plot for the title compound showing (*a*) H⋯H, (*b*) H⋯C, (*c*) O⋯H and (*d*) H⋯F, (*e*) C⋯C and (*f*) H⋯N contacts in detail (cyan dots). The contribution of the inter­actions to the crystal packing amounts to 31.80, 24.30, 15.20, 10.80, 06.20 and 04.30%, respectively. The *d*_e_ (*y* axis) and *d*_i_ (*x* axis) values are the closest external and inter­nal distances (values in Å) from given points on the Hirshfeld surface contacts.

**Figure 7:**
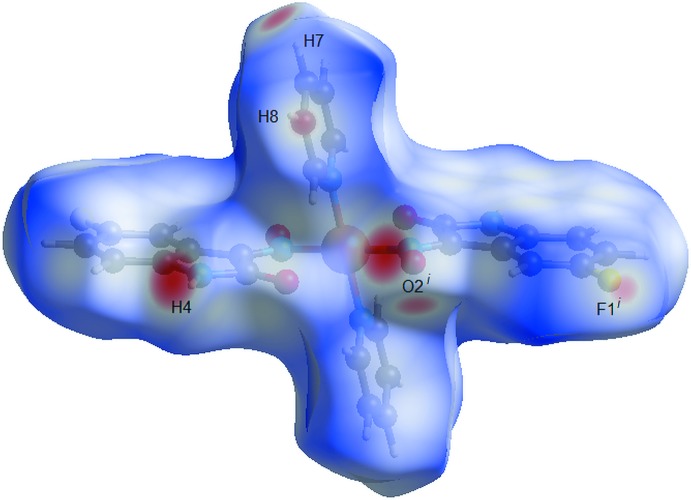
Graphical representation of the Hirshfeld surface (*d*_norm_) for the title compound. The surface is drawn with transparency and simplified for clarity. The surface regions with strongest inter­molecular inter­actions are drawn in magenta and the respective atoms are labelled. [Symmetry code: (i) −*x* + 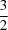, −*y* + 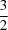, −*z* + 1.]

The Hirshfeld surface analysis (Hirshfeld, 1977 ▸) of the crystal structure suggests that the contributions of the H⋯H, H⋯C and H⋯O inter­molecular inter­actions to the crystal packing amount to 31.80, 24.30 and 15.20%, respectively. Other important inter­molecular contacts for the cohesion of the structure are (values given in %): H⋯F = 10.80, C⋯C = 6.20, and H⋯N = 4.30. The contributions to the crystal cohesion are shown as two-dimensional Hirshfeld surface fingerprint plots with cyan dots (Wolff *et al.*, 2012 ▸). The *d_e_* (*y* axis) and *d_i_* (*x* axis) values are the closest external and inter­nal distances (values in Å) from given points on the Hirshfeld surface contacts (Fig. 6 ▸). The graphical representation of the Hirshfeld surface for the title compound with transparency and labelled atoms (Fig. 7 ▸) indicates, in magenta, the locations of the strongest inter­molecular contacts, *e.g.* the H4, H7, H8, O2 and F1 atoms.

## Database survey   

**Figure 8:**
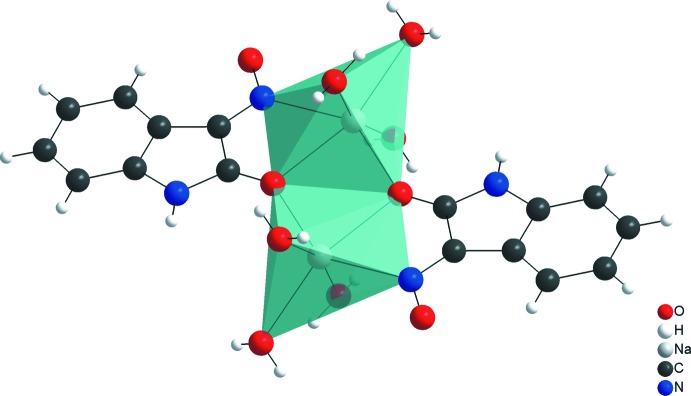
Partial view of the structure of catena-poly[[[aqua­sodium]-di-μ-aqua-[aqua­sodium]-bis­(μ-2-oxoindoline-2,3-dione 3-oximato)] tetra­kis­(oxoindoline-2,3-dione 3-oxime)].

A search of *SciFinder* (SciFinder, 2018 ▸) revealed a single report in the literature about the crystal structure of coordin­ation compounds with isatin 3-oxime derivatives, *i.e.* the one-dimensional coordination polymer, *catena*-poly[[[aquasodium]-di-μ-aqua-[aqua­sodium]-bis­(μ-2-oxoindoline-2,3-dione 3-oximato)] tetra­kis­(oxoindoline-2,3-dione 3-oxime)] (Barreto Martins *et al.*, 2011 ▸). For that complex, the Na cations shows an octa­hedral coordination environment builded by the anionic form of the isatin 3-oxime and water mol­ecules (Fig. 8 ▸).

## Synthesis and crystallization   

All the starting materials were commercially available and were used without further purification. The synthesis of the ligand followed the procedure reported previously (Martins *et al.*, 2017 ▸). 5-Fluoro­isatin 3-oxime was dissolved in methanol (4 mmol, 50 mL) and deprotonated with one pellet of KOH with stirring maintained for 60 min. Simultaneously, a green solution of copper acetate mono­hydrate in methanol (2 mmol, 50 mL) was prepared under continuous stirring. A dark-coloured mixture of both solutions was maintained with stirring at room temperature for 8 h. A crude dark-red material was obtained by evaporation of the solvent. Purple crystals of the complex, suitable for X-ray analysis, were obtained by recrystallization of the solid from a pyridine/methanol (1:10 *v*/*v*) solution.

## Refinement   

Crystal data, data collection and structure refinement details are summarized in Table 2 ▸. Hydrogen atoms were located in a difference-Fourier map, but were positioned with idealized geometry and refined isotropically using a riding model, with *U*_iso_(H) = 1.2*U*_eq_(C, N), and with C—H = 0.95 and N—H = 0.88 Å. 

## Supplementary Material

Crystal structure: contains datablock(s) I, publication_text. DOI: 10.1107/S2056989018003365/rz5228sup1.cif

Structure factors: contains datablock(s) I. DOI: 10.1107/S2056989018003365/rz5228Isup2.hkl

CCDC reference: 1826012

Additional supporting information:  crystallographic information; 3D view; checkCIF report

## Figures and Tables

**Table 1:** Hydrogen-bond geometry (Å, °) *Cg*1 is the centroid of the N3/C9–C13 ring

*D*—H⋯*A*	*D*—H	H⋯*A*	*D*⋯*A*	*D*—H⋯*A*
C9—H5⋯O1^i^	0.95	2.54	3.1424 (18)	121
C12—H8⋯F1^ii^	0.95	2.49	3.287 (2)	142
C13—H9⋯O1	0.95	2.54	3.1077 (19)	119
N1—H4⋯O2^iii^	0.88	2.00	2.7529 (14)	143
C6—H2⋯*Cg*1^iv^	0.95	2.79	3.7076 (17)	162

Symmetry codes: (i) 
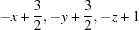
; (ii) 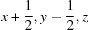; (iii) 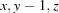; (iv) 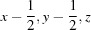.

**Table 2:** Experimental details

Crystal data	
Chemical formula	[Cu(C_8_H_4_FN_2_O_2_)_2_(C_5_H_5_N)_2_]
*M*_r_	580.00
Crystal system, space group	Monoclinic, *C*2/*c*
Temperature (K)	200
*a*, *b*, *c* (Å)	19.9709 (14), 7.2155 (5), 17.1989 (12)
β (°)	98.579 (2)
*V* (Å^3^)	2450.6 (3)
*Z*	4
Radiation type	Mo *K*α
μ (mm^−1^)	0.95
Crystal size (mm)	0.40 × 0.24 × 0.20

Data collection	
Diffractometer	Bruker APEXII CCD
Absorption correction	Multi-scan (*SADABS*; Krause *et al.*, 2015 ▸)
*T*_min_, *T*_max_	0.674, 0.746
No. of measured, independent and observed [*I* > 2σ(*I*)] reflections	18806, 4481, 3966
*R*_int_	0.017
(sin θ/λ)_max_ (Å^−1^)	0.760

Refinement	
*R*[*F* ^2^ > 2σ(*F* ^2^)], *wR*(*F* ^2^), *S*	0.033, 0.086, 1.12
No. of reflections	4481
No. of parameters	178
H-atom treatment	H-atom parameters constrained
Δρ_max_, Δρ_min_ (e Å^−3^)	0.40, −0.34

Computer programs: *APEX2* and *SAINT* (Bruker, 2014 ▸), *SHELXT2014* (Sheldrick, 2015*a* ▸), *SHELXL2016* (Sheldrick, 2015*b* ▸), *WinGX* (Farrugia, 2012 ▸), *DIAMOND* (Brandenburg, 2006 ▸), *CrystalExplorer* (Wolff *et al.*, 2012 ▸), *publCIF* (Westrip, 2010 ▸) and *enCIFer* (Allen *et al.*, 2004 ▸).

## supplementary crystallographic information

### Crystal data

**Table:**

[Cu(C_8_H_4_FN_2_O_2_)_2_(C_5_H_5_N)_2_]	*F*(000) = 1180
*M**_r_* = 580.00	*D*_x_ = 1.572 Mg m^−^^3^
Monoclinic, *C*2/*c*	Mo *K*α radiation, λ = 0.71073 Å
*a* = 19.9709 (14) Å	Cell parameters from 9537 reflections
*b* = 7.2155 (5) Å	θ = 2.4–32.7°
*c* = 17.1989 (12) Å	µ = 0.95 mm^−^^1^
β = 98.579 (2)°	*T* = 200 K
*V* = 2450.6 (3) Å^3^	Prismatic, purple
*Z* = 4	0.40 × 0.24 × 0.20 mm

### Data collection

**Table:**

Bruker APEXII CCD diffractometer	3966 reflections with *I* > 2σ(*I*)
Radiation source: fine-focus sealed X-ray tube, Bruker APEX2 CCD	*R*_int_ = 0.017
φ and ω scans	θ_max_ = 32.7°, θ_min_ = 2.1°
Absorption correction: multi-scan (SADABS; Krause *et al.*, 2015)	*h* = −30→30
*T*_min_ = 0.674, *T*_max_ = 0.746	*k* = −10→7
18806 measured reflections	*l* = −26→26
4481 independent reflections	

### Refinement

**Table:**

Refinement on *F*^2^	Primary atom site location: structure-invariant direct methods
Least-squares matrix: full	Secondary atom site location: difference Fourier map
*R*[*F*^2^ > 2σ(*F*^2^)] = 0.033	Hydrogen site location: inferred from neighbouring sites
*wR*(*F*^2^) = 0.086	H-atom parameters constrained
*S* = 1.12	*w* = 1/[σ^2^(*F*_o_^2^) + (0.035*P*)^2^ + 2.817*P*] where *P* = (*F*_o_^2^ + 2*F*_c_^2^)/3
4481 reflections	(Δ/σ)_max_ < 0.001
178 parameters	Δρ_max_ = 0.40 e Å^−^^3^
0 restraints	Δρ_min_ = −0.34 e Å^−^^3^

### Special details

**Table:**

Geometry. All esds (except the esd in the dihedral angle between two l.s. planes) are estimated using the full covariance matrix. The cell esds are taken into account individually in the estimation of esds in distances, angles and torsion angles; correlations between esds in cell parameters are only used when they are defined by crystal symmetry. An approximate (isotropic) treatment of cell esds is used for estimating esds involving l.s. planes.

### Fractional atomic coordinates and isotropic or equivalent isotropic displacement parameters (Å^2^)

**Table:**

	*x*	*y*	*z*	*U*_iso_*/*U*_eq_	
C1	0.66193 (6)	0.43044 (17)	0.51144 (7)	0.0202 (2)	
C2	0.62981 (6)	0.59529 (16)	0.54136 (7)	0.0179 (2)	
C3	0.57200 (6)	0.53285 (17)	0.57616 (7)	0.0190 (2)	
C4	0.52421 (7)	0.6220 (2)	0.61397 (8)	0.0252 (2)	
H1	0.524139	0.752687	0.620565	0.030*	
C5	0.47665 (7)	0.5092 (2)	0.64150 (10)	0.0308 (3)	
C6	0.47458 (7)	0.3189 (2)	0.63286 (10)	0.0330 (3)	
H2	0.440637	0.248844	0.652851	0.040*	
C7	0.52236 (8)	0.2300 (2)	0.59479 (10)	0.0292 (3)	
H3	0.521610	0.099354	0.587721	0.035*	
C8	0.57102 (6)	0.33887 (17)	0.56761 (8)	0.0213 (2)	
C9	0.79324 (7)	0.8070 (2)	0.66905 (8)	0.0271 (3)	
H5	0.778142	0.929489	0.656022	0.033*	
C10	0.81834 (9)	0.7655 (3)	0.74669 (9)	0.0361 (3)	
H6	0.820299	0.858192	0.786181	0.043*	
C11	0.84039 (8)	0.5880 (3)	0.76578 (9)	0.0374 (4)	
H7	0.857425	0.556392	0.818653	0.045*	
C12	0.83737 (9)	0.4571 (3)	0.70703 (10)	0.0359 (3)	
H8	0.852688	0.334216	0.718775	0.043*	
C13	0.81167 (8)	0.5072 (2)	0.63051 (9)	0.0285 (3)	
H9	0.809607	0.416673	0.590100	0.034*	
Cu1	0.750000	0.750000	0.500000	0.01704 (6)	
F1	0.42924 (6)	0.59199 (17)	0.67886 (8)	0.0505 (3)	
N1	0.62487 (6)	0.28139 (15)	0.52904 (8)	0.0242 (2)	
H4	0.633443	0.165583	0.517849	0.029*	
N2	0.65751 (5)	0.75711 (14)	0.53373 (6)	0.01704 (17)	
N3	0.78961 (5)	0.67952 (17)	0.61181 (6)	0.0208 (2)	
O1	0.71204 (5)	0.42959 (14)	0.47722 (6)	0.02501 (19)	
O2	0.62959 (5)	0.90531 (12)	0.55624 (6)	0.02333 (18)	

### Atomic displacement parameters (Å^2^)

**Table:**

	*U*^11^	*U*^22^	*U*^33^	*U*^12^	*U*^13^	*U*^23^
C1	0.0261 (5)	0.0133 (5)	0.0219 (5)	0.0016 (4)	0.0052 (4)	0.0011 (4)
C2	0.0209 (5)	0.0130 (5)	0.0204 (5)	0.0011 (4)	0.0051 (4)	0.0007 (4)
C3	0.0205 (5)	0.0150 (5)	0.0215 (5)	−0.0005 (4)	0.0041 (4)	0.0022 (4)
C4	0.0254 (6)	0.0219 (6)	0.0299 (6)	0.0018 (5)	0.0092 (5)	0.0011 (5)
C5	0.0246 (6)	0.0335 (8)	0.0367 (7)	0.0022 (5)	0.0131 (5)	0.0036 (6)
C6	0.0254 (6)	0.0329 (8)	0.0420 (8)	−0.0059 (6)	0.0098 (6)	0.0092 (7)
C7	0.0292 (6)	0.0201 (6)	0.0386 (7)	−0.0053 (5)	0.0057 (5)	0.0062 (5)
C8	0.0237 (5)	0.0145 (5)	0.0255 (5)	−0.0007 (4)	0.0036 (4)	0.0033 (4)
C9	0.0295 (6)	0.0297 (7)	0.0220 (6)	0.0023 (5)	0.0034 (5)	−0.0005 (5)
C10	0.0398 (8)	0.0476 (10)	0.0199 (6)	−0.0019 (7)	0.0009 (5)	−0.0030 (6)
C11	0.0345 (7)	0.0532 (11)	0.0230 (6)	−0.0028 (7)	−0.0006 (5)	0.0120 (6)
C12	0.0375 (8)	0.0367 (8)	0.0327 (7)	0.0066 (7)	0.0026 (6)	0.0141 (6)
C13	0.0322 (6)	0.0278 (7)	0.0262 (6)	0.0071 (5)	0.0069 (5)	0.0045 (5)
Cu1	0.01925 (10)	0.01681 (10)	0.01575 (9)	0.00118 (7)	0.00488 (6)	0.00176 (7)
F1	0.0404 (5)	0.0492 (7)	0.0705 (8)	0.0048 (5)	0.0367 (5)	0.0016 (6)
N1	0.0325 (6)	0.0093 (4)	0.0326 (6)	0.0007 (4)	0.0108 (5)	0.0011 (4)
N2	0.0204 (4)	0.0131 (4)	0.0179 (4)	0.0018 (3)	0.0040 (3)	0.0000 (3)
N3	0.0203 (4)	0.0249 (5)	0.0182 (4)	0.0027 (4)	0.0064 (3)	0.0029 (4)
O1	0.0308 (5)	0.0182 (4)	0.0284 (5)	0.0044 (4)	0.0123 (4)	0.0013 (4)
O2	0.0297 (4)	0.0122 (4)	0.0301 (5)	0.0025 (3)	0.0113 (4)	−0.0021 (3)

### Geometric parameters (Å, º)

**Table:**

C1—O1	1.2342 (15)	C9—N3	1.3412 (19)
C1—N1	1.3649 (16)	C9—C10	1.387 (2)
C1—C2	1.4798 (17)	C9—H5	0.9500
C2—N2	1.3070 (15)	C10—C11	1.378 (3)
C2—C3	1.4493 (16)	C10—H6	0.9500
C3—C4	1.3901 (18)	C11—C12	1.378 (3)
C3—C8	1.4072 (17)	C11—H7	0.9500
C4—C5	1.386 (2)	C12—C13	1.387 (2)
C4—H1	0.9500	C12—H8	0.9500
C5—F1	1.3591 (17)	C13—N3	1.3423 (19)
C5—C6	1.382 (2)	C13—H9	0.9500
C6—C7	1.392 (2)	Cu1—N2	2.0176 (10)
C6—H2	0.9500	Cu1—N3	2.0319 (11)
C7—C8	1.3841 (18)	N1—H4	0.8800
C7—H3	0.9500	N2—O2	1.2917 (13)
C8—N1	1.4077 (17)		
			
O1—C1—N1	127.37 (12)	N3—C9—H5	119.0
O1—C1—C2	126.46 (12)	C10—C9—H5	119.0
N1—C1—C2	106.18 (11)	C11—C10—C9	119.13 (15)
N2—C2—C3	133.98 (11)	C11—C10—H6	120.4
N2—C2—C1	118.12 (10)	C9—C10—H6	120.4
C3—C2—C1	107.89 (10)	C10—C11—C12	119.00 (14)
C4—C3—C8	120.61 (12)	C10—C11—H7	120.5
C4—C3—C2	133.96 (12)	C12—C11—H7	120.5
C8—C3—C2	105.40 (11)	C11—C12—C13	119.13 (15)
C5—C4—C3	116.20 (13)	C11—C12—H8	120.4
C5—C4—H1	121.9	C13—C12—H8	120.4
C3—C4—H1	121.9	N3—C13—C12	122.02 (15)
F1—C5—C6	118.42 (13)	N3—C13—H9	119.0
F1—C5—C4	117.69 (14)	C12—C13—H9	119.0
C6—C5—C4	123.89 (14)	N2—Cu1—N3	88.75 (4)
C5—C6—C7	119.82 (13)	O1—Cu1—N3	89.01 (4)
C5—C6—H2	120.1	C1—N1—C8	110.49 (10)
C7—C6—H2	120.1	C1—N1—H4	124.8
C8—C7—C6	117.53 (13)	C8—N1—H4	124.8
C8—C7—H3	121.2	O2—N2—C2	120.09 (10)
C6—C7—H3	121.2	O2—N2—Cu1	124.18 (8)
C7—C8—C3	121.93 (13)	C2—N2—Cu1	115.18 (8)
C7—C8—N1	128.03 (12)	C9—N3—C13	118.66 (12)
C3—C8—N1	110.04 (11)	C9—N3—Cu1	119.59 (10)
N3—C9—C10	122.06 (15)	C13—N3—Cu1	121.74 (10)
			
O1—C1—C2—N2	−2.32 (19)	C4—C3—C8—N1	−178.83 (12)
N1—C1—C2—N2	177.94 (12)	C2—C3—C8—N1	−0.40 (14)
O1—C1—C2—C3	179.09 (13)	N3—C9—C10—C11	0.1 (2)
N1—C1—C2—C3	−0.65 (14)	C9—C10—C11—C12	0.5 (3)
N2—C2—C3—C4	0.5 (3)	C10—C11—C12—C13	−0.6 (3)
C1—C2—C3—C4	178.76 (14)	C11—C12—C13—N3	0.0 (2)
N2—C2—C3—C8	−177.63 (14)	O1—C1—N1—C8	−179.33 (13)
C1—C2—C3—C8	0.63 (13)	C2—C1—N1—C8	0.41 (14)
C8—C3—C4—C5	−0.3 (2)	C7—C8—N1—C1	179.97 (14)
C2—C3—C4—C5	−178.20 (14)	C3—C8—N1—C1	−0.01 (16)
C3—C4—C5—F1	179.91 (13)	C3—C2—N2—O2	−4.6 (2)
C3—C4—C5—C6	−0.4 (2)	C1—C2—N2—O2	177.27 (11)
F1—C5—C6—C7	179.92 (15)	C3—C2—N2—Cu1	167.30 (11)
C4—C5—C6—C7	0.2 (3)	C1—C2—N2—Cu1	−10.83 (14)
C5—C6—C7—C8	0.6 (2)	C10—C9—N3—C13	−0.7 (2)
C6—C7—C8—C3	−1.3 (2)	C10—C9—N3—Cu1	178.48 (12)
C6—C7—C8—N1	178.68 (14)	C12—C13—N3—C9	0.6 (2)
C4—C3—C8—C7	1.2 (2)	C12—C13—N3—Cu1	−178.54 (12)
C2—C3—C8—C7	179.62 (13)		

### Hydrogen-bond geometry (Å, º)

Cg1 is the centroid of the N3/C9–C13 ring

**Table:**

*D*—H···*A*	*D*—H	H···*A*	*D*···*A*	*D*—H···*A*
C9—H5···O1^i^	0.95	2.54	3.1424 (18)	121
C12—H8···F1^ii^	0.95	2.49	3.287 (2)	142
C13—H9···O1	0.95	2.54	3.1077 (19)	119
N1—H4···O2^iii^	0.88	2.00	2.7529 (14)	143
C6—H2···*Cg*1^iv^	0.95	2.79	3.7076 (17)	162

Symmetry codes: (i) −*x*+3/2, −*y*+3/2, −*z*+1; (ii) *x*+1/2, *y*−1/2, *z*; (iii) *x*, *y*−1, *z*; (iv) *x*−1/2, *y*−1/2, *z*.

## References

[bb1] Allen, F. H., Johnson, O., Shields, G. P., Smith, B. R. & Towler, M. (2004). *J. Appl. Cryst.* **37**, 335–338.

[bb2] Barreto Martins, B., Bresolin, L., Santana Carratu, V., Boneberger Behm, M. & Bof de Oliveira, A. (2011). *Acta Cryst.* E**67**, m790–m791. 10.1107/S1600536811018290 PMC3120341 21754670

[bb3] Brandenburg, K. (2006). *DIAMOND*. Crystal Impact GbR, Bonn, Germany.

[bb4] Bruker (2014). *APEX2*, *SADABS* and *SAINT*. Bruker AXS Inc., Madison, Wisconsin, USA.

[bb5] Erdmann, O. L. (1841*a*). *Ann. Chim. Phys.* **3**, 355–371.

[bb6] Erdmann, O. L. (1841*b*). *J. Prakt. Chem.* **22**, 257–299.

[bb7] Farrugia, L. J. (2012). *J. Appl. Cryst.* **45**, 849–854.

[bb8] Hirshfeld, H. L. (1977). *Theor. Chim. Acta*, **44**, 129–138.

[bb9] Krause, L., Herbst-Irmer, R., Sheldrick, G. M. & Stalke, D. (2015). *J. Appl. Cryst.* **48**, 3–10. 10.1107/S1600576714022985 PMC4453166 26089746

[bb10] Laurent, A. (1841). *Ann. Chim. Phys.* **3**, 371–383.

[bb11] Martins, B. B., Bresolin, L., Farias, R. L. de, Oliveira, A. B. de & Gervini, V. C. (2017). *Acta Cryst.* E**73**, 987–992. 10.1107/S2056989017008301 PMC5499275 28775867

[bb12] Meleddu, R., Distinto, S., Corona, A., Tramontano, E., Bianco, G., Melis, C., Cottiglia, F. & Maccioni, E. (2017). *J. Enzyme Inhib. Med. Chem.* **32**, 130–136. 10.1080/14756366.2016.1238366 PMC6010014 27766892

[bb13] Mishra, P., Kumar, A., Mamidi, P., Kumar, S., Basantray, I., Saswat, T., Das, I., Nayak, T. K., Chattopadhyay, S., Subudhi, B. B. & Chattopadhyay, S. (2016). *Sci. Rep.* **6**, 20122. 10.1038/srep20122 PMC4740769 26843462

[bb14] SciFinder (2018). Chemical Abstracts Service: Columbus, OH, 2010; RN 58-08-2 (accessed Feb 08, 2018).

[bb15] Sheldrick, G. M. (2015*a*). *Acta Cryst.* A**71**, 3–8.

[bb16] Sheldrick, G. M. (2015*b*). *Acta Cryst.* C**71**, 3–8.

[bb17] Sumpter, W. C. (1944). *Chem. Rev.* **34**, 393–434.

[bb18] Westrip, S. P. (2010). *J. Appl. Cryst.* **43**, 920–925.

[bb19] Wolff, S. K., Grimwood, D. J., McKinnon, J. J., Turner, M. J., Jayatilaka, D. & Spackman, M. A. (2012). *CRYSTAL EXPLORER*. University of Western Australia, Perth, Australia.

